# COVID-19 vaccine reluctance and possible driving factors: A comparative assessment among pregnant and non-pregnant women

**DOI:** 10.3389/fpubh.2022.1100130

**Published:** 2023-01-09

**Authors:** Erum Rehman, Nadia Rehman, Muhammad Akhlaq, Iftikhar Hussain, Ondrej Holy

**Affiliations:** ^1^School of Economics, Shandong University of Finance and Economics, Jinan, China; ^2^Department of Mathematics, COMSATS University, Islamabad, Pakistan; ^3^Department of Pharmacy, Hazara University, Mansehra, Pakistan; ^4^Department of Mathematical Sciences, Karakoram International University Gilgit, Gilgit, Pakistan; ^5^Science and Research Center, Faculty of Health Sciences, Palacký University Olomouc, Olomouc, Czechia

**Keywords:** vaccine hesitancy, pregnant women, vaccination, COVID-19, VAX scale

## Abstract

The coronavirus disease of 2019 (COVID-19) constitutes a serious threat to pregnant women. One of the key strategies for preventing and managing the COVID-19 epidemic is vaccination. Herd immunity is significantly hampered by COVID-19 vaccine reluctance, which poses a potential threat to population health. Therefore, the present work intends to ascertain the incidence and severity of COVID-19 vaccine hesitancy among Pakistani pregnant women, the determinants driving their decision, and a comparative assessment with non-pregnant participants. This cross-sectional survey was carried out from November 2021 to February 2022. The validated vaccination attitude examination (VAX) scale about vaccination reluctance was undertaken by participants, who were also required to indicate whether they would be inclined to acquire the COVID-19 vaccine along with the reasons for reluctance. In comparison to the non-pregnant category with 353 participants, the group of 372 pregnant participants who responded to the questionnaire had a much greater proportion of hesitant respondents. Likewise, contrasted to 31% of non-pregnant participants, about 40% of them attributed their willingness to get vaccinated against coronavirus to social media. They also demonstrated a considerably stronger mean score on all subcategories of the VAX measure. The adjusted odd ratio findings showed that the independent factors for vaccine reluctance appeared to be trusting rumors on social media (adj OR: 2.58), not being afraid of covid-19 (adj OR: 2.01), not believing in COVID-19 existence (adj OR: 2.53), and not believing in vaccines (adj OR: 4.25). Uncertainty about the COVID-19 vaccine is very prevalent among expectant mothers. The investigation accentuates the pressing need to administer COVID-19 vaccination to the general public, including expectant mothers who might be anxious about the vaccine.

## Introduction

Thousands of casualties have occurred as a result of the coronavirus disease pandemic of 2019 (COVID-19), which has also triggered public health challenges, overwhelmed healthcare infrastructures, disrupted supply chains, and the economic systems, and ended up causing a mental illness epidemic (1–3). Epidemiological evidence demonstrated that expectant women have a higher tendency for contracting COVID-19 infection (4, 5). The rate of hospitalization in intensive care departments and the need for mechanical respiration have been reported far higher in pregnant women than in non-pregnant women. They are also more susceptible to dying from COVID-19 and encounter challenges during pregnancy, such as premature delivery (6, 7). Differential stages of anxiety and depression were also found linked to the ongoing pandemic (8), significantly raising the incidence of pregnancy-related abnormalities like preeclampsia, nausea, vomiting, distress, low birth weight, premature births, and low Apgar scores (9). Henceforth, COVID-19 vaccinations for pregnant women are timely warranted to combat the unpleasant outcomes (10, 11). Unfortunately, this vulnerable population was not enrolled in any of the COVID-19 vaccination trials that have been conducted so far. Also, there is a lack of evidence and some degree of ambiguity regarding the COVID-19 vaccine's consequences on gestation (12, 13).

A widely productive public health approach for preventing contagious diseases is immunization, which has been demonstrated to drastically lower infection-related morbidity and mortality worldwide (14, 15). While vaccines are widely adopted, it is feasible to limit and even eliminate diseases that can be prevented by vaccines. Consequently, in order to manage these infections, a significant immunization rate must be attained. The World Health Organization's Strategic Advisory Group of Experts on Immunization (SAGE) working group defines vaccine hesitancy as postponing or rejecting immunization services irrespective of their availability which hampers efforts to combat diseases that can be prevented through vaccination (16, 17). In accordance with all applicable legislation, several COVID-19 vaccines have been manufactured and approved for use in the wider population across the world. In the COVID-19 vaccination developmental phases, pregnant women were not included though, they have had access to all the manufactured vaccines after the FDA allowed the inoculation of Pfizer/BioNTech for the pregnant subjects at the beginning of 2021. The European Medicines Agency then responded in a similar manner (18, 19). The American College of Obstetricians and Gynecologists (20) and the Society for Maternal-Fetal Medicine have persistently espoused the accessibility of the COVID-19 vaccine to expectant and breastfeeding mothers, and both healthcare communities, along with the Center for Disease Control (CDC), presently propose inoculation of coronavirus vaccine for this population (21). This recommendation was based on the grounds that none of the proposed vaccines consist of activated viruses or adjuvants- that may endanger an unborn fetus.

Several absurd conspiracy ideas emerged on media platforms as soon as COVID-19 was declared a plague. Such conspiracy theories have caused polio vaccination campaigns to fail in Pakistan, a country that is particularly susceptible to them. Following the epidemic, rumors began to emerge in the country that the COVID-19 vaccine is a hoax and part of a conspiracy against Muslim nations. This notion, which supports COVID-19 vaccination reluctance, was widely debated in the local communities. It is believed that COVID-19 infection in Pakistan may lead to several complications in expectant women who have not gotten vaccinated. It was discovered that COVID-19 had an 8% mortality rate in pregnant women when local data was gathered and discussed at a webinar on the COVID-19 vaccine among pregnant women conducted by a public medical university in collaboration with the American Society of Microbiology (22–24). These unfavorable pregnancy risks highlight the significance of vaccinating expectant mothers. In recent investigations, the effectiveness of the COVID-19 vaccines in the population of pregnant women has been evaluated, and the findings have been encouraging. According to UK research, immunizations did not affect perinatal outcomes since women who got at least one shot of the COVID-19 vaccine in the time of pregnancy had equal rates of all unfavorable pregnancy results to unimmunized women (25, 26). Additionally, recommendations for immediate vaccination of expectant women have been established, emphasizing that the vaccine's advantages outweigh any possible risks (27). Pregnant women are not allowed to participate in clinical trials, which makes it challenging for them to develop faith in vaccinations and has hampered the practice of vaccinating pregnant women. Moreover, the effectiveness of the vaccination campaign for pregnant women has been further hampered by the widespread conspiracy beliefs in Pakistan concerning vaccination campaigns, which claim that they are a part of a Western stratagem to render Muslim women sterile (23, 28).

Henceforth, related institutions and healthcare professionals must identify vaccine hesitancy and its causes to implement targeted measures to reduce it and increase vaccine uptake, particularly during the times of pandemic when the willingness of pregnant women to acquire the vaccination may get affected. Owing to the paucity of evidence on the acceptance, awareness, and constraints of vaccination among pregnant women in the COVID-19 context, we carried out a hospital-based cross-sectional survey among pregnant and non-pregnant participants in a public hospital in Pakistan to evaluate the determinants that influence vaccine acceptability the acceptance of influenza vaccination and associated factors in the context of COVID-19 pandemic.

## Materials and methods

### Data source and study population

From November 2021 to February 2022, cross-sectional research was carried out in the outpatient setting of the Obstetrics and Gynecology department of the Benazir Bhutto Hospital affiliated with the Rawalpindi Medical University, Rawalpindi, Pakistan.

To obtain data more conveniently, an Urdu-translated VAX (Vaccination Attitude Examination) index was designed to assess anti-vaccination beliefs, and it has been verified in 2017 (29). The questionnaire consisted of four sub-categories with a total of 12 items. A scale of 1–6 is used to score each item, with 1 denoting a “strongly disagree” response and 6 denoting a “strongly agree” response. When examining vaccination intentions for COVID-19 infection, prior research has demonstrated that the subject survey approach has a high degree of reliability (30). Overall, a higher VAX score indicates a more intense level of anti-vaccine attitude. The VAX scales may be further divided into categories based on the item numbers: items # 1–3 deal with mistrust of vaccine benefits, items # 4–6 deal with the concerns about unforeseen future effects, items # 7–9 deal with commercial profit concerns, and item# 10–12 deal with the preference for natural immunity.

During the study period, the optimal sample size was estimated to be at least 369, employing a convenient sampling approach with ~5% marginal error at a 95% confidence level and a 60% estimated vaccination rate. Among 416 study participants who consented to partake in the survey and respond to the questionnaire, 44 were disqualified owing to incomplete survey responses, retaining 372 pregnant individuals. For non-pregnant women participants, the sample size was estimated following similar criteria. To evaluate if pregnancy exclusively is the primary rationale for denying the COVID-19 vaccination, a similar survey was given to 399 reproductive-age non-pregnant women (in the outpatient department), and 353 of them responded. With a total of 725 responses, the present investigation comprised both pregnant and non-pregnant women of reproductive age who had not received the COVID-19 vaccine during the research period. Respondents may review a questionnaire that was virtually identical to the paper form in terms of queries, language, and presentation chronology to maintain physical distance and avoid the spread of coronavirus infection. Study participants were provided web links to questionnaires by the research team, but each participant was only permitted to electronically respond to a single query since the electronic database's content sorting process was fully mechanized. The survey questionnaire was designed to gather data on the demographic trends of the respondents, comprising age, place of origin, marital status, income level, educational attainment, and occupational status. By adding a few more items to the VAX survey, we also asked respondents about their comfort with the COVID-19 vaccination and other common vaccines. These additional questions included not believing in vaccines, believing in social media rumors, prior unpleasant vaccine side effects, receiving inadequate evidence about vaccines, not being afraid of COVID-19, not believing in the existence of SARS-CoV-2, and not being afraid of COVID-19. All items in the questionnaire included a categorical “Yes” or “No” response option.

### Ethical considerations

Each study subject completed a written informed consent form after receiving a detailed description of the research's objectives and potential outcomes. Our study was carried out in accordance with the Benazir Bhutto Hospital's Scientific Ethics Committee and the Helsinki Declaration Guidelines for Human Subjects in Scientific research (*Ref: BBH-2022/006052*).

### Statistical analysis

Descriptive and inferential statistics were performed using IBM SPSS software, version 26.0. The mean (*X*) and standard deviation (SD) were adopted to illustrate continuous data, whilst absolute values and percentages (%) were utilized to represent categorical variables. The average values of the data evaluated in the investigation were compared using the ANOVA and the Student's *t*-testing. Applying the non-parametric SPSS Median test and the pregnancy status as the grouping variable, the findings of the VAX scale were given as median values and interquartile range (IQR). The Pearson correlation coefficient was adopted to assess parametric data, whereas Spearman's correlation coefficient was determined for non-parametric variables. In a multivariate backward stepwise logistic regression model, all variables determined in the univariate investigation to have a statistically significant relationship with vaccination reluctance were included. The Chi-square and Fisher's exact tests were used to compare the proportions of reluctant, uncertain, and confident respondents. The level of significance for statistical analysis was set at α = 0.05.

## Results

Overall, 372 pregnant and 353 non-pregnant women of childbearing age answered the survey's questionnaires. The findings demonstrated comparable outcomes in terms of age (32.5 ± 8.6 vs. 29.6 ± 8.9), place of origin (In rural case: 43.28% vs. 41.36%; In urban case: 56.72% vs. 58.64%), marital status (Married: 93.82% vs. 90.37%; single/divorced/widowed: 6.18% vs. 9.63%), and educational level (undergraduate: 25.81% vs. 29.18%; graduate: 51.61% vs. 55.81%). However, the similarities were not relatively significant. Given the higher ratio of pregnant participants who were unemployed (21.1% vs. 11.2%, with *p* = 0.012), we discovered that participants who were pregnant had much lower income levels than their non-pregnant counterparts (58.33% below average income, vs. 50.01%, with *p* = 0.011). Further, both groups of participants showed comparable opinions about trusting the COVID-19 vaccination (pregnant cases: 33.33% vs. non-pregnant cases: 30.31%) and other vaccines (pregnant cases: 82.53% vs. non-pregnant cases: 89.52%), with around 67% of all the pregnant participants not trusting the COVID-19 vaccinations and ~69.99% in non-pregnant cases (Table 1).

**Table 1:** Baseline characteristics of pregnant and non-pregnant study participants.

**Characteristics**	**Pregnant women *n* = (372)**	**Non-pregnant women *n* = (353)**	***p*-Value**
Age	32.5 ± 8.6	29.6 ± 8.9	0.073
**Place of origin**		
Rural	161 (43.28)	146 (41.36)	0.652
Urban	211 (56.72)	207 (58.64)	
**Marital status**		
Married	349 (93.82)	319 (90.37)	0.164
Single/widowed/divorced	23 (6.18)	34 (9.63)	
**Income level**		
Below average	217 (58.33)	180 (50.01)	0.011
Above average	155 (41.66)	173 (49.01)	
**Educational attainment**		
Undergraduate	96 (25.81)	103 (29.18)	0.707
Graduate	192 (51.61)	197 (55.81)	
Master and above	84 (22.58)	53 (15.01)	
**Occupational status**		
Employed	284 (76.34)	308 (87.25)	0.013
Unemployed	88 (23.66)	45 (12.75)	
**Trusting SARS-CoV-2 vaccine**		
Yes	124 (33.33)	107 (30.31)	0.401
No	248 (66.67)	246 (69.69)	
**Trusting other vaccines**		
Yes	307 (82.53)	316 (89.52)	0.421
No	65 (17.47)	37 (10.48)	

p < 0.05 is considered significant.

The VAX questionnaire and six additional items about variables that might influence COVID-19 vaccination denial were distributed among the study's pregnant and non-pregnant subjects. Table 2 represents the outcomes of VAX questionnaire assessments and the vaccine hesitancy reasons of the selected study participants. We found that pregnant participants exhibited considerably greater levels of distrust in the vaccinations against COVID-19 infection (32 vs. 28 with *p* < 0.001) while comparing the overall VAX average score. In every category of the VAX survey's questions, pregnant study participants outscored non-pregnant study participants. More precisely, compared to the other group, pregnant participants expressed greater levels of negative attitudes against immunizations in essence (VAX item# 1–3). In comparison to non-pregnant subjects, pregnant participants expressed greater concerns about unforeseen vaccination adverse outcomes (VAX item #4–6), held more unfavorable attitudes on COVID-19 vaccines (VAX item #7–9), and showed increased health knowledge (VAX item#10–12). Broadly speaking, the VAX survey revealed that considerably pregnant cases are much more apprehensive than non-pregnant cases (51.34% vs. 38.24%, with *p* < 0.001). Contrarily, non-pregnant participants were more inclined to have reservations about receiving the vaccination (32.29% vs. 7.80%). Further, we also discovered that, of the six survey questions that addressed concerns about vaccinations that weren't covered by the VAX survey, believing rumors on social media showed the strongest effect on hesitation to get vaccinated. Approximately 40% of the pregnant cases responded positively to those questions compared to 31.16% of the non-pregnant participants.

**Table 2:** VAX assessments and reasons for the hesitancy of the study participants.

	**Pregnant women *n* = (372)**	**Non-pregnant women *n* = (353)**	***p*-Value**
**VAX items score, Median (IQR)**	32 (9)	28 (10)	**0.001**
VAX items# 1–3 (mistrust of vaccine benefit)	8 (4)	6 (4)	**0.001**
VAX items# 4–6 (worries over unforeseen future effects)	12 (7)	10 (6)	0.012
VAX item# 7–9 (concerns about commercial profit)	7 (4)	5 (3)	**0.001**
VAX item# 10–12 (preference to natural immunity)	9 (5)	8 (5)	0.011
**COVID-19 vaccination feeling**			
Confident	152 (40.86%)	104 (29.46%)	**0.001**
Unsure	29 (7.80%)	114 (32.29%)	
Hesitant	191 (51.34%)	135 (38.24%)	
**Other reasons for hesitancy**			
Trusting rumors on social media	147 (39.52%)	110 (31.16%)	0.029
Previous unpleasant side effects	87 (23.39%)	75 (21.25%)	0.403
Insufficient information about vaccines	56 (15.05%)	70 (19.83%)	0.663
Not afraid of COVID-19	46 (12.37%)	51 (14.45%)	0.322
Not believing in SARS-CoV-2 existence	20 (5.38%)	19 (5.38%)	0.022
Not believing in vaccines	16 (4.30%)	28 (7.93%)	0.054

Bold values represent significance at < 0.05.

Table 3 summarizes the variations among pregnant study participants depending on the other reasons for hesitancy criteria i.e., decision factors. We discovered that out of the 372 pregnant women, 152 (40.86%) were confident, 29 (7.80%) were unsure, and 191 (51.34%) were hesitant about getting the COVID-19 vaccination. Among 195 (52.42%) cases, believing in social media rumors was the most prevalent trigger. While compared to confident pregnant study subjects (57.24%), hesitant pregnant study subjects (63.35%) showed statistically significant higher trust in social media. Furthermore, having previously experienced unpleasant vaccine adverse reaction was a key contributor to COVID-19 vaccination hesitation. Only 28 (7.52%) pregnant participants identified this rationale, while 17.24% of the pregnant participants were unsure.

**Table 3:** Assessment of pregnant study participants based on decision factors.

**Decision factors**	**Overall *n* = (372)**	**Confident *n* = (152)**	**Unsure *n* = (29)**	**Hesitant *n* = (191)**	***p*-Value**
Trusting rumors on social media	195 (52.42%)	87 (57.24%)	17 (58.62%)	121 (63.35%)	**0.001**
Previous unpleasant side effects	28 (7.52%)	11 (7.24%)	3 (10.34%)	5 (2.62%)	**0.000**
Insufficient information about vaccines	57 (15.32%)	21 (13.82%)	5 (17.24%)	28 (14.66%)	0.349
Not afraid of COVID-19	31 (8.33%)	10 (6.58%)	2 (6.90%)	19 (9.95%)	0.653
Not believing in SARS-CoV-2 existence	28 (7.53%)	11 (7.24%)	1 (3.45%)	7 (3.66%)	0.408
Not believing in vaccines	33 (8.87%)	12 (7.89)	1 (3.45%)	11 (5.76%)	0.307

Bold values represent significance at < 0.05.

Table 4 summarizes the outcomes of the risk factor analysis of pregnant study participants against COVID-19 vaccine hesitancy. The risk variables we discovered for vaccination reluctance in pregnant and non-pregnant study participants were rural background, poor income, embracing social media rumors, not trusting the occurrence of coronavirus disease or vaccinations, and not being scared of COVID-19 infection. Furthermore, the hesitation was exacerbated by prior exposure to adverse vaccination effects, but only in non-pregnant participants. Last but not least, it was discovered that trusting the COVID-19 vaccine served as a resilience factor against immunization reluctance in expectant mothers [OR = 0.55, CI (0.31–1.46)].

**Table 4:** Risk factor analysis of pregnant women against COVID-19 vaccination.

	**Hesitancy against COVID-19 vaccination**			
**Risk factors**	**Pregnant**	***p*** **-Value**	**Non-pregnant**	***p*** **-Value**
	**OR (95% CI)**		**OR (95% CI)**	
Age	0.96 (0.88–1.13)	0.604	0.99 (0.92–1.12)	0.623
**Place of origin**				
Rural	1.89 (1.21–2.07)	0.010	1.72 (1.13–2.02)	0.021
Urban	1.28 (0.96–1.92)		1.10 (0.85–1.20)	
**Marital status**				
Married	0.76 (0.39–1.14)	0.365	0.93 (0.76–1.16)	0.302
Single/widowed/divorced	0.99 (0.48–1.28)		0.87 (0.63–1.04)	
**Income level**				
Below average	2.68 (1.88–3.25)	0.001	2.99 (2.01–3.86)	0.003
Above average	1.34 (1.01–1.76)		1.11 (0.87–1.73)	
**Educational attainment**				
Undergraduate	1.17 (0.91–1.67)	0.524	1.26 (0.89–1.92)	0.503
Graduate	1.23 (0.87–1.56)		1.34 (1.00–1.77)	
Master and above	1.02 (0.72–1.54)		1.14 (0.82–1.87)	
**Occupational status**				
Employed	1.19 (0.92–1.54)	0.112	1.01 (0.91–1.35)	0.124
Unemployed	1.52 (0.98–2.14)		1.49 (1.02–1.86)	
**Reasons for hesitancy**				
Trusting rumors on social media	3.29 (2.56–4.08)	0.001	2.89 (2.19–3.96)	0.001
Previous unpleasant side effects	1.46 (1.12–1.93)	0.051	1.56 (1.09–2.06)	0.031
Insufficient information about vaccines	2.16 (1.87–2.87)	0.109	2.09 (1.92–2.68)	0.201
Not afraid of COVID-19	2.78 (2.01–3.33)	0.000	3.13 (2.86–4.11)	0.000
Not believing in SARS-CoV-2 existence	3.64 (2.64–5.21)	0.000	3.29 (2.82–5.01)	0.000
Not believing in vaccines	5.18 (3.31–7.29)	0.001	6.43 (4.21–9.36)	0.001
**Trusting SARS-CoV-2 vaccine**				
Yes	0.55 (0.31–1.46)	0.033	0.63 (0.23–2.01)	0.054
No	1.28 (0.91–2.98)		1.21 (0.95–2.86)	
**Trusting other vaccines**				
Yes	0.91 (0.38–1.27)	0.062	1.13 (0.67–1.78)	0.043
No	1.66 (1.05–2.36)		1.83 (1.12–2.75)	

p < 0.05 is considered significant.

OR, odds ratio; CI, confidence interval.

We discovered that the rural settings were a negligible independent risk factor (adjusted OR:1.47 with *p*-value = 0.053) after controlling for risk variables linked to vaccine hesitation among the group of pregnant participants. According to the outcomes based on the odd ratio analysis, the statistically significant independent risk variables were not being afraid of COVID-19 infection, having a poor income status, trusting social media rumors, not believing the coronavirus disease existence, and not trusting the vaccines (Table 5).

**Table 5:** Analysis for adjusted odd ratios of the potential factors associated with hesitancy among pregnant women.

**Reasons for hesitancy**	**Adjusted OR (95% CI)**	***p*-Value**
Rural place of origin	1.47 (0.87–2.15)	0.483
Below-average level income	2.13 (1.45–2.77)	0.122
Trusting rumors on social media	2.58 (2.09–3.22)	**0.000**
Not afraid of COVID-19	2.01 (1.76–2.56)	**0.010**
Not believing in SARS-CoV-2 existence	2.53 (2.01–3.12)	**0.000**
Not believing in vaccines	4.25 (2.68–6.43)	**0.000**

Bold values represent significance at < 0.05.

OR, odds ratio; CI, confidence interval.

## Discussion

For all we know, the present work offers significant evidence of the underlying causes of COVID-19 vaccine reluctance among pregnant women in the Pakistani populace. Inconsistent with prior evidence, perceptions against COVID-19 vaccination have been more negative than those of other vaccine-preventable diseases. In comparison to developed nations, the acceptability of the COVID-19 vaccine among healthcare professionals is relatively lower in emerging economies than it is for the seasonal influenza vaccine (31). According to a recent survey conducted in the United States, 20% of Canadians and 25% of Americans exhibited a propensity to decline the coronavirus vaccine, which is congruent with generalized resistance to immunizations (32). Likewise, uncertainties about the COVID-19 vaccine's safeness, effectiveness, and usefulness were the main reasons why nurses in Hong Kong were reluctant to receive it, yet concerns about the flu vaccine's importance were the primary grounds (33).

Pregnant women's intention to acquire the COVID-19 vaccine and its major contributors were investigated in a multi-national, cross-sectional study by disseminating an online questionnaire across some European economies (Belgium, Ireland, Norway, the Netherlands, Switzerland, and the United Kingdom) in the first wave of the pandemic. Low-educated and unemployed participants had a lower likelihood of receiving the COVID-19 vaccine (34). A cross-sectional online questionnaire was performed in sixteen regions to assess the acceptability level of COVID-19 vaccination among pregnant women along with their prospective determinants. Approximately 52% of the participants who were pregnant planned to get the vaccination, anticipating that it could be 90% efficacious. The most important determinants of acceptance of the COVID-19 vaccine included feeling more confident in the reliability and safety of the COVID-19 vaccine and regular vaccines, understanding the importance of vaccination, having concerns about COVID-19, adhering to COVID-19 guidelines, and having faith in the public health care system (35). It can be suggested that the information sources had a significant influence on the apprehensive decisions of pregnant participants since they were more inclined to consider social media sources. This result is found aligned with another work done in Italy with similar findings (36). The study discovered that sources of information and confidence in healthcare professionals are the most important determinants of the acceptability of mandatory vaccination.

We exclusively evaluated vaccination reluctance in women in our investigation, therefore the influence of gender on vaccine reluctance could not be identified. However, the female gender has been identified as a significant predictor of vaccine hesitancy in a wide range of investigations (37–40). Consequently, the coronavirus outbreak has highlighted the necessity for addressing the gender disparity in vaccine aversion, which has mostly been disregarded, except for pregnant cases. Conforming to an investigation on sexual differences in vaccine hesitancy, men were reported more inclined to undergo coronavirus vaccines (41, 42). A research investigation conducted on six hundred and seventy-two study participants exhibited a ratio of 67% to the corona vaccination acceptability overall whereas elderly, males, Asians, and graduates were more inclined toward getting vaccinated when compared to their peers (43). Conversely, considerable demographics and geographical discrepancies in the COVID-19 vaccine acceptance were discovered, stressing the importance of evidence-based community outreach to encourage acceptance and combat the pandemic. Taking into consideration these outcomes and doubts regarding the COVID-19 rapid assessment and authorization procedures, the most subsequent conclusion may be understood (44). Particularly, the rapid manufacture of the vaccine against COVID-19, which might lead to vaccine reluctance in both populations, overall and pregnant cases, may account in part for the discrepancies in perceptions regarding immunizations overall and the corona vaccination in particular. The digital and social networking channels, including Facebook and Instagram the most popular social media platforms in Pakistan, allow anti-vaccination activists to promote false facts despite institutional backing for the COVID-19 vaccine's efficiency and tolerability.

In our investigation, pregnant participants appeared to be more impacted by social factors including education and income level, along with misleading facts from social networks than other groups that were susceptible to the vaccination against COVID-19 infection. In line with other research done in various regions (45, 46), our research demonstrated that poorer educational and income levels were significant predictors of vaccine hesitancy (47, 48). Contrary to other findings on vaccination, this association suggests that people with higher levels of education and income are more concerned about the efficacy and safety of vaccines (49, 50).

The breadth of the present investigation is confined to a population-based survey. Consequently, the outcomes may not be comparable to women in other communities, where reliance on electronic media and uptake of COVID-19 vaccinations could vary considerably. Rural background, poor income status, and inadequate educational attainment are additional country-based attributes that may influence vaccine reluctance. These attributes are more frequent in the Pakistani population than in those of other developing economies. Further limitations include an online survey approach and insufficient sample size estimation for each category.

## Conclusion

Based on the considerable evidence supporting the safety of the COVID-19 vaccine, the overall population, including expectant mothers who could have concerns related to vaccine safety, urgently needs the vaccination against COVID-19. In Pakistan, pregnant women are reluctant to receive vaccinations due to the significant part played by social and electronic networking. Therefore, we recommend the enactment of targeted measures to combat reluctant factors as well as policy instruments that approach pregnant women to promote COVID-19 immunization.

## Data availability statement

The raw data supporting the conclusions of this article will be made available by the authors, without undue reservation.

## Ethics statement

This survey study was approved by the Ethics Committee of Benazir Bhutto Hospital, Punjab Province, Pakistan (Ref: BBH-2022/006052). All procedures were conducted following the guidelines of our institutional Ethics Committee and in compliance with the principles of the Declaration of Helsinki. Informed consent was obtained from all subjects involved in the study. The patients/participants provided their written informed consent to participate in this study.

## Author contributions

Conceptualization and supervision: ER and OH. Methodology: ER, NR, and IH. Software, formal analysis, data curation, and preparation: ER and NR. Validation: ER, NR, and MA. Investigation: ER, NR, MA, and IH. Writing original draft: ER, NR, MA, IH, and OH. Writing, review, and editing: NR, MA, IH, and OH. Visualization: ER. All authors have read and agreed to the final version of the manuscript.
